# Can we predict necrosis intra-operatively? Real-time optical quantitative perfusion imaging in surgery: study protocol for a prospective, observational, in vivo pilot study

**DOI:** 10.1186/s40814-017-0204-1

**Published:** 2017-11-25

**Authors:** Sanne M. Jansen, Daniel M. de Bruin, Mark I. van Berge Henegouwen, Simon D. Strackee, Denise P. Veelo, Ton G. van Leeuwen, Suzanne S. Gisbertz

**Affiliations:** 1 Academic Medical Center, University of Amsterdam, Dep. of Biomedical Engineering & Physics, Meibergdreef 9, 1105 AZ Amsterdam, The Netherlands; 2 Academic Medical Center, University of Amsterdam, Dep. of Plastic, Reconstructive and Handsurgery, Meibergdreef 9, 1105 AZ Amsterdam, The Netherlands; 3 Academic Medical Center, University of Amsterdam, Dep. of Gastrointestinal Surgery, Meibergdreef 9, 1105 AZ Amsterdam, The Netherlands; 4 Academic Medical Center, University of Amsterdam, Dep. of Anesthesiology, Meibergdreef 9, 1105 AZ Amsterdam, The Netherlands

**Keywords:** Optical technologies, Optical coherence tomography, Sidestream darkfield microscopy, Laser speckle contrast imaging, Fluorescence imaging, Perfusion, Monitoring, Necrosis, Anastomotic leakage, Feasibility, Accuracy, Risk prediction

## Abstract

**Background:**

Compromised perfusion as a result of surgical intervention causes a reduction of oxygen and nutrients in tissue and therefore decreased tissue vitality. Quantitative imaging of tissue perfusion during reconstructive surgery, therefore, may reduce the incidence of complications. Non-invasive optical techniques allow real-time tissue imaging, with high resolution and high contrast. The objectives of this study are, first, to assess the feasibility and accuracy of optical coherence tomography (OCT), sidestream darkfield microscopy (SDF), laser speckle contrast imaging (LSCI), and fluorescence imaging (FI) for quantitative perfusion imaging and, second, to identify/search for criteria that enable risk prediction of necrosis during gastric tube and free flap reconstruction.

**Methods:**

This prospective, multicenter, observational in vivo pilot study will assess tissue perfusion using four optical technologies: OCT, SDF, LSCI, and FI in 40 patients: 20 patients who will undergo gastric tube reconstruction after esophagectomy and 20 patients who will undergo free flap surgery. Intra-operative images of gastric perfusion will be obtained directly after reconstruction at four perfusion areas. Feasibility of perfusion imaging will be analyzed per technique. Quantitative parameters directly related to perfusion will be scored per perfusion area, and differences between biologically good versus reduced perfusion will be tested statistically. Patient outcome will be correlated to images and perfusion parameters. Differences in perfusion parameters before and after a bolus of ephedrine will be tested for significance.

**Discussion:**

This study will identify quantitative perfusion-related parameters for an objective assessment of tissue perfusion during surgery. This will likely allow early risk stratification of necrosis development, which will aid in achieving a reduction of complications in gastric tube reconstruction and free flap transplantation.

**Trial registration:**

Clinicaltrials.gov registration number NCT02902549. Dutch Central Committee on Research Involving Human Subjects registration number NL52377.018.15.

## Background

In vivo imaging plays an important role at all stages of the health care cycle, e.g., in screening, staging, and treatment selection and during response monitoring. The key technology for personalized patient monitoring is multidimensional quantitative imaging, combining longitudinal information from multiple modalities at a macroscopic and microscopic level.

At present, surgeons lack validated imaging tools to visualize and quantitatively evaluate microcirculation response in real-time during surgery. Decreased perfusion results in lack of oxygen and nutrients in tissue and therefore can lead to ischemia and necrosis development [1]. Because of a lack in perfusion monitoring, complications due to vascular compromise are a major problem, especially in reconstructive surgery. If, however, changes in perfusion could be monitored and risk of necrosis development could be predicted intra-operatively, then surgeons could change their reconstructive design and the anesthesiologists could improve perfusion with the administration of fluids, inotropes, or vasopressors, if necessary [2].

In this study, we focus on two major reconstructive interventions: gastric tube reconstruction and free flap surgery, because of their high social and clinical impact.

Anastomotic leakage after esophagectomy is the most feared complication in gastrointestinal surgery. The incidence is high (2.5–20%) and development of strictures (30–42%) and mortality (2–3%) are the results of this complication [3, 4]. Multiple risk factors have been studied for anastomotic leakage development, such as age, nutritional state, and radiation, but the major contributing factor of leakage development is compromised perfusion [1]. Since the incidence of esophageal cancer is rising [5], the problem is growing.

In free flap surgery, total flap loss of transplanted flaps occurs in 4.4–10% [6], but many more patients develop partial necrosis. Perfusion monitoring is important in reconstructive surgery, and numbers of flap complications are rising with a higher incidence in oncologic reconstructions (4.5 million in the USA in 2015) [7].

Surgeons need a tool that allows imaging with a high resolution (microcirculation), direct (intra-operative), in 3D (to image thrombosis, luminal narrowing, or distinct overlaying vessels), and that produces quantitative data to objectively interpret such images.

Optical techniques, whose contrast is based on the interaction of light with tissue, are able to image tissue at a high resolution and in real-time [8–11]. Tissues change in different (surgical) settings; thus, with the help of optical techniques, we expect we will be able to evaluate tissue changes intra-operatively.

Some of these techniques are FDA-approved and have emerged as powerful diagnostic tools in different departments of medicine, such as ophthalmology for visualizing the retina and dermatology for skin cancer diagnostics [12, 13]. For most technologies however, clinical implementation has not been achieved yet due to lack of validation. Because it is unclear which perfusion-related parameter is the most predictive for tissue necrosis development, we want to compare four techniques: optical coherence tomography (OCT), sidestream darkfield microscopy (SDF), laser speckle contrast imaging (LSCI), and fluorescence imaging (FI).

Following phase 1 of the IDEAL recommendations [14], the objectives of this study are, first, to assess the feasibility and accuracy of these four novel optical technologies: OCT, SDF, LSCI, and FI for quantitative perfusion imaging, and, second, to identify/search for criteria that allow risk prediction of necrosis during gastric tube and free flap reconstruction.

## Methods/Design

### Ethical consideration

Methodology was developed based on the STROBE guideline [15] and the STARD [16] statement. This study is approved by the medical ethical committee of the Academic Medical Center, Amsterdam (2015_057). The protocol is registered by The Dutch Central Committee on Research Involving Human Subjects (NL52377.018.15). Also, this study is submitted to the clinicaltrials.gov database (NCT02902549).

All patients will receive patient information both verbally and written by the clinical researcher. Before surgery, written informed consent will be obtained in the case of patient approval. This study will be conducted based on the Good Clinical Practice Guidelines [17] and Principles of the Declaration of Helsinki [18].

### Study design

This ongoing study is a prospective, observational, in vivo, pilot study of 40 patients undergoing reconstructive surgery in either the Gastrointestinal Surgery Department or the Plastic, Reconstructive, and Hand Surgery Department of the Academic Medical Center, University of Amsterdam. During gastric tube reconstruction after esophagectomy as well as free flap reconstruction, images and measurements of (reconstructive) tissue perfusion will be obtained by the four optical techniques: OCT, SDF, LSCI, and FI.

With OCT and SDF, we will image four perfusion areas: from the supplying artery towards the tip of the tissue (also, from good to reduced perfusion), with the hypothesis that perfusion parameters will decrease towards the fundus. With LSCI and FI, we will image the total area of the gastric tube or flap, before and after a bolus of ephedrine 5 mg, which increases blood pressure and cardiac output.

During the operation, hemodynamic parameters (blood pressure, cardiac output, and heart rate), ventilator parameters (spO_2_, minute volume, FiO_2_, tidal volume, and respiratory rate), and medication information at the timing of monitoring will be collected. After surgery, images will be analyzed and scored on quality and perfusion-related parameters (explained below under primary outcome parameters a–e). Moreover, accuracy of the perfusion parameters will be judged based on the distinctiveness between different perfusion areas. To obtain patient outcome data, follow-up of patients will be performed, based on the definitions used in the ECCG (comorbidities, complications, laboratory results, CT/endoscopy/reoperation in patients at risk) at 3 months after surgery. Also, adverse events (AE) will be reported at The Dutch Central Committee on Research Involving Human Subjects.

### Study objectives

#### Primary objective

- To assess the feasibility of OCT, SDF, LSCI, and FI as a quantitative perfusion imaging modality, during gastric tube and free flap reconstruction in terms of image quality, clinical use, and time consumption.
- To test and develop quantitative perfusion (related) parameters which can be used to distinguish between the four perfusion areas (from good to reduced perfusion) and to identify differences in parameters before and after an increase in blood pressure and cardiac output.
- To test the impact of noise levels on the accuracy of perfusion-related parameters measured with LSCI and FI.
- To explore the correlation between the quantitative parameters obtained during surgery and patient outcome in terms of risk assessment of tissue necrosis and anastomotic leakage, defined by the ECCG classification and diagnosed by visualization of flap necrosis development and in case of anastomotic leakage CT scanning of the thorax/abdomen with contrast, upper GI endoscopy, drainage of saliva/gastric content via wound/drains, and/or by reoperation [19].
- To identify criteria for early risk assessment of necrosis.

#### Secondary objectives

- To compare the four imaging modalities in terms of resolution, imaging depth, and field of view.

### Participants

This study will enroll both male and female patients, meeting the following inclusion criteria: above 18 years of age and scheduled for either free flap reconstruction or esophagectomy with gastric tube reconstruction. Moreover, only patients who are informed about the study prior to surgery and who gave written informed consent at least a week before surgery will be included. Exclusion criteria are (because of ICG injection for FI imaging) allergy to iodide, hyperthyroidism, and breastfeeding. Patients who meet any of the exclusion criteria will not be enrolled in this study.

### Sample size calculations

In this study, we will include patients undergoing gastric tube or free flap reconstruction and we will analyze the primary outcomes separately for both groups. For the sample size calculation, we focused on the accuracy with which changes in hemodynamics can be detected. We will report Hedges’ *g* effect size [20] as a measure of the relative magnitude of change in patients undergoing each type of surgery. We will calculate Hedges’ *g* as the mean difference between hemodynamic values before and after surgery divided by the pooled standard deviation based on measurements made before and after surgery.

A sample size of 20 patients per type of surgery will have 80% power to detect a Hedges’ *g* effect size of 0.66, using a paired *t* test with a 0.05 two-sided significance level. An effect size of 0.66 can be arbitrarily defined as between medium and large [21]. As we will include patients undergoing two types of surgery, we will include a total of 2 × 20 = 40 patients in the study.

In addition, this sample size is sufficient for the reliability analysis. A sample size of 20 patients per type of surgical treatment achieves 90% power to detect an intra-class correlation of 0.80 (“substantial agreement”) under the alternative hypothesis when the intra-class correlation under the null hypothesis is 0.40 (“fair agreement”), using an *F* test with a two-sided significance level of 0.05 [22, 23].

The primary outcome parameters will be:

- The feasibility of OCT, SDF, LSCI and FI to qualitatively image perfusion during reconstructive surgery.
- The feasibility of the quantitative perfusion related parameters (items (a)–(e), below) to measure differences of perfusion before and after ephedrine and between four perfusion areas by determining:
  - Vessel density and decorrelation measured with OCT
  - Velocity, microvascular flow index, total vessel density, perfused vessel density measured with SDF
  - Perfusion units measured with LSCI
  - Time constant and time to peak measured with FI
  - Noise influence on perfusion-related parameters measured with all techniques (explained in the last paragraph in the “Procedure” section)
- Patient outcome defined by the ECCG classification in terms of necrosis diagnosed by visualization (in case of flap reconstruction) or anastomotic leakage identified by CT scanning of the thorax/abdomen with contrast, upper GI endoscopy, drainage of saliva/gastric content via wound/drains, and/or by reoperation [19] (in case of gastric tube reconstruction).
- The difference in quantitative perfusion-related parameters between necrosis/leakage and no-necrosis/leakage development.

Secondary outcome parameters will be resolution, imaging depth, and field of view of the four different techniques.

### Procedure

**Fig. 1:**
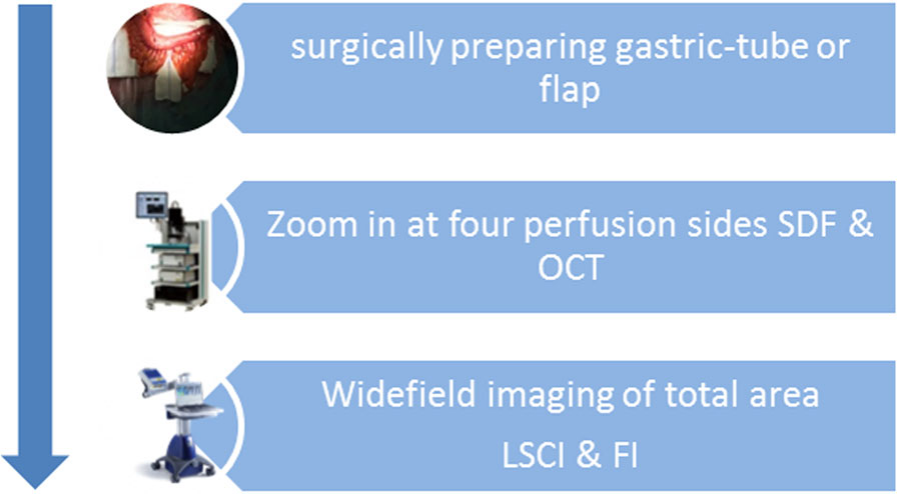
Study protocol flowchart

In both gastric tube reconstruction and free flap reconstruction, intra-operative images will be obtained directly after construction of the gastric tube or elevation of the flap (Fig. 1).

First, we will utilize the Santec OCT system (Santec OCT system IVS-2000, Santec USA Corporation, Hackensack, USA). OCT is a handheld, non-contact, medium field of view (1 × 1–20 × 20 mm), near infrared (1310 nm), and depth-resolved imaging modality that creates images in 3D [24]. We will zoom in at a FOV of 10 mm × 10 mm and 2 mm in depth. The axial resolution of the system is 12 μm, and the lateral resolution is 50 μm. Before we will start imaging the four perfusion areas, a sterile drape (C-arc) is placed over the OCT handheld probe and fibers. The system will be placed at the tissue using a sterile attachment of 6 cm on the lens to create optimal focus distance. Three-dimensional images of 10 mm (*x*, length) by 10 mm (*y*, width) by 4 mm (*z*, depth) will be obtained from the four perfusion areas (1024, 1024, 400 pixels). Also, M-mode scans, point scans in depth at the same location in time, will be obtained with the settings 0 mm (*x*, length) by 10 mm (*y*, width) by 4 mm (*z*, depth) with, respectively, 0, 1024, and 1024 pixels. Motion of particles (red blood cells) within the field of view changes the OCT signal. The change of the OCT signal in time can be expressed as the decorrelation time *τ* (ms). The inverse decorrelation time is proportional to the velocity of the particles and can be used to quantitatively measure perfusion [13]. A faster decorrelation time will be measured with an increased motion of particles, thus in the case of good perfusion.

After OCT measurements, SDF measurements will be carried out with the Microscan (Microvision Medical, Amsterdam, the Netherlands). SDF is a handheld, in-contact, small field of view (0.94 mm × 0.75 mm), green light (530 nm) imaging system that zooms in on the microcirculation. Image resolution is high, and red blood cells are visible flowing through capillaries. Image contrast is based on the absorption of green light by the oxyhemoglobin in RBCs [10]. Therefore, RBCs will appear as dark spots on a bright background. Before we will start imaging the four perfusion areas, a sterile drape (video-camera drape) will be placed over the SDF handheld probe and fibers. A disposable cap will be placed over the ring light camera. A custom-made SDF stabilizer of stainless steel will be used to decrease image drifting due to peristalsis and patient heartbeat and breathing. This SDF stabilizer creates adhesion of the tissue to the tip of the SDF by negative pressure and is validated to be used in patients for the measurements of perfusion [25]. Data will be analyzed using the AVA3.2 software, and perfusion-related parameters will be measured in terms of microvascular flow index (MFI), total vessel density (TVD), perfused vessel density (PVD), proportion of perfused vessels (PPV), de Backer score (DBS), and velocity (μm/s). Velocity will be measured by movement of RBCs which can be plotted in a space-time diagram [26]. The DBS is the vessel density obtained by placing a grid of three equidistant horizontal and three equidistant vertical lines over the SDF image. The number of grid crossings divided by the total length of the grid lines is the de Backer score [27].

Next, LSCI images (MoorFLPI-1™, Moor Instruments, Devon, UK) will be made of the total area of the gastric tube or flap. LSCI is a non-contact, wide field of view (adjustable between 5 mm × 7 mm and 15 cm × 20 cm), near-infrared system, operating at a wavelength of 785 nm [28]. This imaging modality measures speckle fluctuations which are created by the motion and number of particles (RBCs) [29]. We will make 10 LSCI images at two exposure times: 4.0 and 8.3 ms, to find out which setting has the highest sensitivity of microcirculation. This will be done before and after ephedrine administration. The system will be placed 40 cm above the patient.

Finally, the FI of the total area will be performed with the Artemis system (Quest Medical Imaging, Middenmeer, the Netherlands). FI is a non-contact, wide field of view (15 cm × 20 cm), near-infrared (795 nm) imaging modality [11]. This technique is able to visualize the influx of an exogenous fluorophore. In this study, we will use the fluorophore indocyanine green (ICG) to image perfusion influx in the gastric tube or free flap. ICG is widely used in ophthalmology for retinal vascularization imaging and is FDA-approved. The camera will be placed 40 cm above the gastric tube or flap, so the total target tissue is visible in the field of view. After starting the camera for imaging, 2.5 mL ICG will be given intravenously by the anesthesiologist. After 1.5 min, we will administer 5 mg ephedrine to evaluate whether there is a change in perfusion parameters and to test the capability of LSCI and FI to register changes in perfusion. After plotting the intensity of ICG increase in a region of interest (ROI) over time, we will measure the linear part of the rise versus time, called time constant (the ICG concentration versus time is expected to behave as a [1 - exp(time/timeconstant)] function). Moreover, we will measure the time that it takes to come to the maximum value of ICG intensity: the time-to-peak.

In the wide field of view techniques, LSCI and FI, noise levels will be measured in a ROI without perfusion (using a metric ruler placed on the stomach or flap tissue) to obtain data on the sensitivity and false positive perfusion measurements of both techniques.

### Image analysis

Feasibility analyses (primary objective): two independent observers (SMJ, DMdB) will score all stored images with regard to image quality and quantitative parameters of vessel density and decorrelation (OCT), microvascular flow index, total vessel density, perfused vessel density, proportion of perfused vessels and blood flow velocity (SDF), flux (LSCI) and ICG intensity/time, and time constant and time to peak (FI). The type of perfusion parameters depend on the specific characteristics of the imaging modality (e.g., field of view, resolution, imaging depth). Images will be presented to the observers in a random order, and both observers will be blinded to the intraoperative time periods in which the images were obtained.

**Fig. 2:**
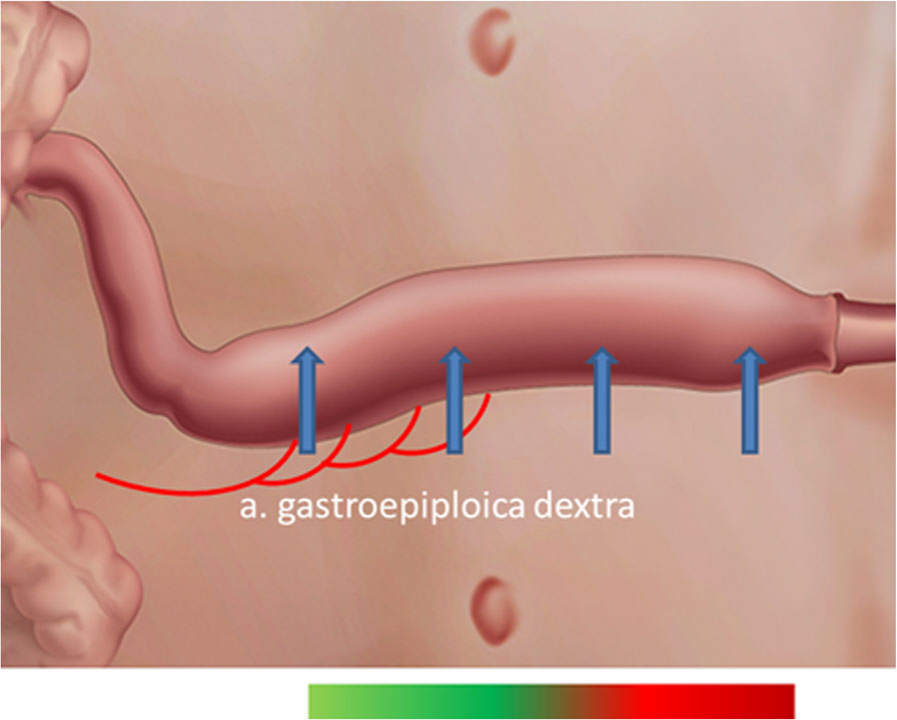
Gastric tube with four image areas and perfusion bar: good-reduced

**Fig. 3:**
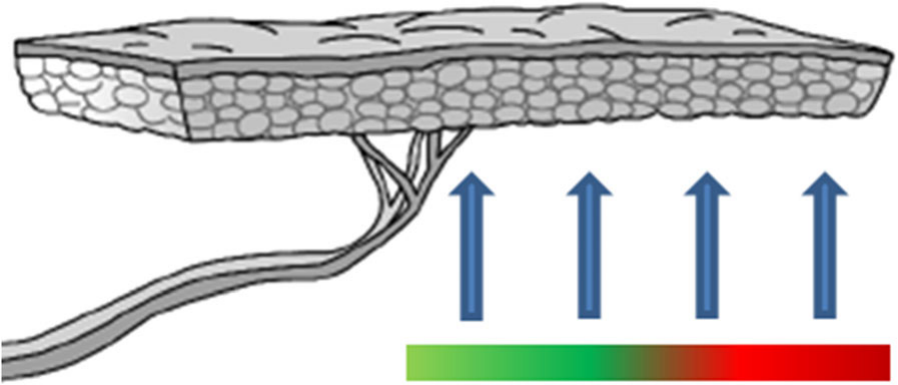
Flap with four image areas and perfusion bar: good-reduced [37]

Accuracy analyses (primary objective): In the surgical interventions, we test the following biological hypotheses: in gastric tube reconstruction after esophagectomy, the hemodynamic perfusion parameters will significantly decrease at the fundus of the stomach after vessel ligation, compared to the antrum of the stomach near the right gastroepiploic artery (Fig. 2); in free flap surgery, the hemodynamic perfusion parameters will significantly decrease from the supplying artery towards the tip of the flap (Fig. 3). Perfusion will significantly improve after the administration of 5 mg ephedrine.

### Statistical analysis

Statistical analysis will be based on a per-protocol approach. Patient baseline characteristics and imaging characteristics will be summarized using simple descriptive statistics.

In the *feasibility analysis*, we will analyze the feasibility of the different techniques to image perfusion intra-operatively. Two independent observers will score all the images on the aspects of light, stability and focus, and hemodynamic indices. Agreement between the experts will be analyzed using the intra-class correlation coefficient. In the *accuracy analysis*, the difference between mean hemodynamic values (before and after ephedrine) will be analyzed using a paired *t* test. Differences will also be defined in Hedges’ *g* effect sizes. The repeated data structure of the four perfusion areas will be additionally analyzed using linear mixed models. In all analyses, statistical uncertainty will be expressed in 95% confidence limits.

Technical comparison of the four imaging modalities in terms of resolution, imaging depth, and field of view will be described in quantitative terms. In view of the explorative nature of this study, we will not adjust for multiple comparisons.

## Discussion

Before starting this in vivo study during surgery, the four imaging modalities were validated for perfusion measurements. This study has been completed in a laboratory setting, using a tissue-like flow phantom [30]. Briefly, the results show the feasibility of OCT, SDF, and LSCI to image perfusion in real-time, high resolution, and high contrast. Moreover, differences between velocity settings from 20 to 0 mm/s could be assessed in perfusion-related parameters with all techniques, which actually gives support to the viability of including early risk prediction of tissue necrosis as a study objective.

Our aim, to test the clinical feasibility of and compare OCT, SDF, LSCI, and FI, will delay the operation by approximately 20 min. However, this delay was well accepted by the surgeons by recognizing the potentially colossal clinical implications that these methods may have for identifying strategies that allow intra-operative risk prediction of necrosis. Even with only 20 patients included per therapy, we expect we can measure at least a trend in the correlation of patient outcome and measurements of perfusion with OCT, SDF, LSCI, and FI. In the scope of this study, we will provide essential answers in terms of technique feasibility and accuracy for quantitative perfusion measurements and monitoring in surgery with the aim to create fundaments for future research in the IDEAL 2a phase.

This is the first study in which OCT is used for intra-operative perfusion measurements in gastric tube reconstruction after esophagectomy. OCT started off as an imaging method in ophthalmology, and research currently extends to other fields such as cardiology, urology, dermatology, and oncology. Wong et al. successfully visualized subsurface blood vessels in a skin flap of a rat with color Doppler OCT [31]. Our study will show the feasibility of OCT to image perfusion in surgery, the system handling by the surgeons, and the data processing, accuracy, and risk stratification of obtained perfusion parameters.

SDF is developed for sublingual tissue evaluation but was previously tested in skeletal muscle and small intestine serous surface in a rat model by Turek et al. [32]. Also, de Bruin et al. evaluated the role of SDF in bowel perfusion diagnostics during gastrointestinal surgery [33]. SDF was feasible to image bowel tissue, but differences between perfusion areas were not assessed. In our study, we will compare different perfusion areas in terms of microvascular flow index, proportion of perfused vessels, perfused vessel density, total vessel density, de Backer score, and velocity.

LSCI was previously described to evaluate perfusion in the gastric tube of an animal model by Klijn et al. [34], showing a decrease in perfusion units towards the fundus. Milstein et al. recently published the evaluation of gastric tube microcirculation with LSCI, showing differences in perfusion before and after reconstruction and in reverse Trendelenburg [35]. We will compare flux measurements obtained at two integration times (4.0 ms/8.3 ms) and measure differences between them. Moreover, we will look at the difference in flux between the four perfusion areas and between patients who develop necrosis or leakage compared with patients who do not.

FI was recently qualitatively evaluated in the prediction of anastomotic leakage suggesting an important diagnostic role for patients undergoing gastric tube reconstruction [36]. In that study, 144 patients were enrolled and a correlation was seen between the perfusion areas (good vs. sparse perfused) and development of anastomotic leakage. In our study, we will develop FI in a diagnostic tool that measures quantitative data to objectively image perfusion intra-operatively, i.e., by calculating perfusion-related parameters in terms of the ICG intensity/time and the time to peak.

In all techniques, noise will be measured and scored in our perfusion images and the influence of noise on perfusion-related parameters will be measured and described.

## Conclusion

This study will identify quantitative perfusion-related parameters for an objective assessment of tissue perfusion during surgery. This may allow early risk stratification of necrosis development, which will aid in achieving a reduction of complications in gastric tube reconstruction after esophagectomy and free flap transplantations.

### Trial status

Open, patients enrolling.

## Abbreviations

AE: Adverse events
FI: Fluorescence imaging
FiO_2_: Fraction of inspired oxygen
FOV: Field of view
ICG: Indocyanine green
LSCI: Laser speckle contrast imaging
OCT: Optical coherence tomography
RBC: Red blood cell
SDF: Sidestream darkfield microscopy
spO_2_: Peripheral capillary oxygen saturation
STARD: Standards for Reporting of Diagnostic Accuracy
STROBE: Strengthening the Reporting of Observational Studies in Epidemiology
